# Graphene removal by water-assisted focused electron-beam-induced etching – unveiling the dose and dwell time impact on the etch profile and topographical changes in SiO_2_ substrates

**DOI:** 10.3762/bjnano.15.18

**Published:** 2024-02-07

**Authors:** Aleksandra Szkudlarek, Jan M Michalik, Inés Serrano-Esparza, Zdeněk Nováček, Veronika Novotná, Piotr Ozga, Czesław Kapusta, José María De Teresa

**Affiliations:** 1 Academic Centre for Materials and Nanotechnology, AGH University of Krakow, av. A. Mickiewicza 30, 30-059 Krakow, Poland; 2 Department of Solid State Physics, Faculty of Physics and Applied Computer Science, AGH University of Krakow, av. A. Mickiewicza 30, 30-059 Krakow, Poland; 3 Instituto de Nanociencia y Materiales de Aragón (INMA), CSIC-Universidad de Zaragoza, 50009 Zaragoza, Spain; 4 NenoVision s.r.o. Purkyňova 649/127, 612 00 Brno, Czech Republic; 5 Department of Power Electrical and Electronic Engineering, Faculty of Electrical Engineering and Communication, Brno University of Technology, Technická 3082/12, Královo Pole, 61600, Brno, Czech Republic; 6 Institute of Metallurgy and Materials Science, Polish Academy of Sciences, 25 Reymonta Street, 30-059 Krakow, Poland; 7 Departamento de Física de la Materia Condensada, Universidad de Zaragoza, 50009 Zaragoza, Spain; 8 Laboratorio de Microscopías Avanzadas (LMA), Universidad de Zaragoza, E-50018 Zaragoza, Spain

**Keywords:** direct writing, dwell time, electron dose, etching, graphene, maskless lithography, nanopatterning

## Abstract

Graphene is one of the most extensively studied 2D materials, exhibiting extraordinary mechanical and electronic properties. Although many years have passed since its discovery, manipulating single graphene layers is still challenging using standard resist-based lithography techniques. Recently, it has been shown that it is possible to etch graphene directly in water-assisted processes using the so-called focused electron-beam-induced etching (FEBIE), with a spatial resolution of ten nanometers. Nanopatterning graphene with such a method in one single step and without using a physical mask or resist is a very appealing approach. During the process, on top of graphene nanopatterning, we have found significant morphological changes induced in the SiO_2_ substrate even at low electron dose values (<8 nC/μm^2^). We demonstrate that graphene etching and topographical changes in SiO_2_ substrates can be controlled via electron beam parameters such as dwell time and dose.

## Introduction

The discovery of extraordinary and controllable electrical conductivity in graphene back in 2004 made it the most recognized 2D material [1]. The newly discovered phenomena, such as unconventional strong electron–electron interactions, present in superlattices formed in twisted bilayered and trilayered graphene, led to the emergence of a new field called “twistronics” – just to highlight recent remarkable discoveries such as superconductivity [2–3], topological phases [4–5], and the unusual hydrodynamic behavior of electrons, which was observed in narrow graphene nanoconstrictions [6]. Given these unique properties, it is unsurprising that graphene became a top candidate for a broad range of applications in optoelectronics and possible future energy-efficient and high-speed communication devices. All those future technologies will require high-precision lithography techniques with excellent lateral resolution, high throughput, and minimized possibility of material damage. In the last decade, several approaches have been made to provide the most suitable method for patterning graphene films, each with its own set of advantages and disadvantages. Most of the current techniques are based on multistep processing. For example, ultranarrow graphene nanoribbons can be formed with the so-called meniscus-mask lithography [7] or nanospheres lithography [8], although positioning and shape control are very limited in those cases. Conventional electron beam lithography (EBL) reaches the resolution of a few nanometers. However, it leaves residual resists on the surface [9], which strongly affects electrical transport properties [10]. A similar high resolution can be achieved with e-beam bombardment, which initially introduces defects into the graphene structure and then knocks out carbon atoms, although the edges of the fabricated nanostructures remain rough after the process [11]. Other direct techniques, such as focused ion beam (FIB) milling with heavy Ga^+^ ions, are not applicable due to the high impact on the underlying substrate. Helium ion milling was believed to be the most suitable tool for structuring graphene [12]. However, it requires expensive equipment, and even this technique introduces a substantial number of defects into the graphene layer, as shown by Kim et al. [13].

A direct graphene etching was proposed using a thin ice layer on top of the graphene surface. Upon interaction with electrons, the ice is dissociated into the reactive ions H^+^ or OH^−^, which subsequently interact with carbon atoms and form volatile species [14]. This method is modified based on the direct delivery of water molecules into the scanning electron microscope chamber. This process is called focused electron-beam-induced etching (FEBIE) and was already demonstrated for thin amorphous carbon membranes a decade ago [15]. Oxygen or water vapor can be used for etching graphene [16–17] and all carbon allotropes, such as diamond [18–19] or carbon nanotubes [20]. Although the fundamentals of the FEBIE method are easily intelligible, the process includes complex surface kinetics phenomena occurring between electrons and adsorbed molecules [21]. Hence, the resolution of the method is dependent on the precursor dynamics (adsorption/desorption rate, diffusion), electron beam (lateral size, electron flux, energy), and scanning parameters (dwell time, refresh time, scanning strategy) [22]. Additionally, residual hydrocarbons inside the scanning electron microscope chamber manifest as an unwanted co-deposition of amorphous carbon. Those deposition and etching processes may co-exist and can be controlled to a certain level by the electron flux [23]. The influence of surface kinetics phenomena on the etch profiles has not been considered in the previous works describing the proof of concept of water-assisted graphene etching. Moreover, the effects of changes in the topography of Si/SiO_2_ substrate during this process have not been addressed so far, as they may be observed only under certain experimental conditions.

In a standard scanning electron microscope, the morphological changes upon stationary exposure were investigated in the work of Stevens Kalceff et al. [24], where the authors observed an increase/decrease in volume of crystalline and amorphous SiO_2_, respectively. However, the explicit mechanism responsible for the process that considers possible reactions paths with the dissociated products of residual water molecules was not provided.

An emission of charged Si and O ions from the SiO_2_ surface upon electron exposure was studied in the 1990s. This phenomenon, known as electron-stimulated desorption (ESD) was observed in UHV conditions by Baragiola et al. [25] and Chen et al. [26] as an effect of the interaction with strong trapped charges (holes), although under much higher electron doses (>3 × 10^6^ nC/μm^2^) or beam currents (> μA) compared to those used in our studies.

For this work, we selected high-quality graphene, mechanically exfoliated onto a SiO_2_/Si substrate, which contains a low amount of defects as described elsewhere [27].

In the first part of the present contribution, we demonstrate how the beam parameters and the dose affect the etched profiles and consequently the lateral resolution of water-assisted FEBIE of graphene. The Raman analysis provides information about the degree of damage caused by this method. Atomic force microscopy (AFM) measurements reveal important aspects of topographical changes induced in the substrate and help to establish optimized conditions for the etching process.

## Results

The fundamentals of water-assisted FEBIE have already been schematically shown in the graphical abstract. They can be described as follows: the scanning electron microscope chamber is filled with water molecules, which physisorb onto the graphene. When the beam locally dissociates adsorbed molecules, the reactive species (H^+^ or OH^−^) are formed, and they start to chemically react with graphene. There are a few possible reaction paths which could occur during this process:


[1] $${\text{C}}_{\left(\text{ads}\right)}+4{\text{H}}^{+}\to {\text{CH}}_{4}$$

[2] $${\text{C}}_{\left(\text{ads}\right)}+{\text{OH}}^{-}\to \text{CO}+1/2{\text{H}}_{2}$$

[3] $${\text{C}}_{\left(\text{ads}\right)}+2{\text{OH}}^{-}\to {\text{CO}}_{2}+{\text{H}}_{2}$$

**Figure 1:**
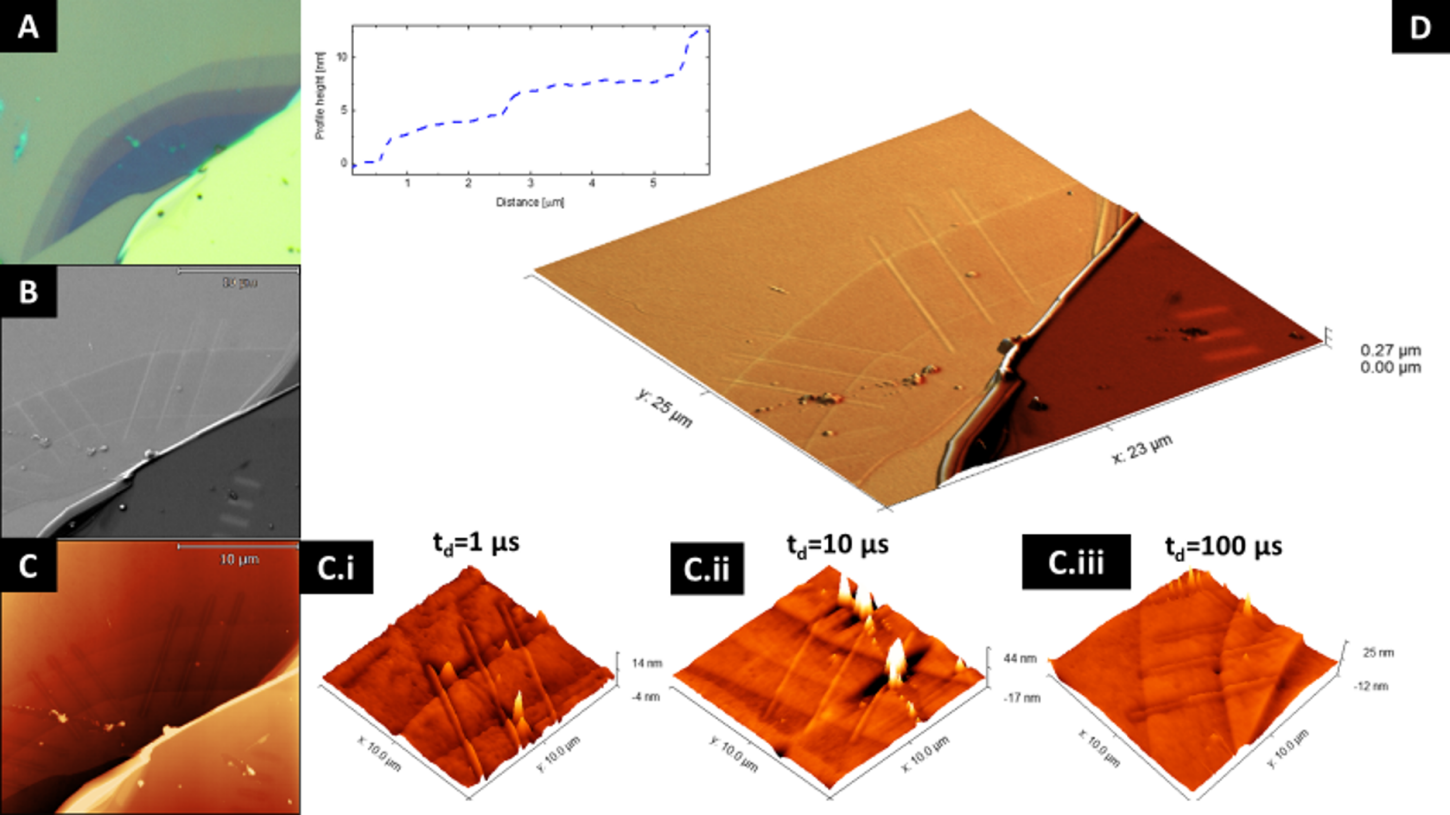
Graphene flake composed of monolayer, bilayer, and triple-layer graphene after water-assisted etching processes, analyzed using different microscopy methods: A) optical microscopy; B) SEM; C) AFM of the lines etched using different dwell times: i) 1 μs, ii) 10 μs, and iii) 100 μs. D) Correlative probe and electron microscopy composed from the correlated signals of SEM and AFM analysis. The inset shows the AFM scan profile over monolayer, bilayer at triple-layer graphene.

A first series of water FEBIE experiments have been performed on single, bi-, and triple layers of graphene in ESEM mode at a background pressure of 130 Pa, with a beam current of 4 nA and a beam energy of 5 kV using the selected values of the dwell times (1 μs, 10 μs, 100 μs) and doses (400 nC/μm^2^, 200 nC/μm^2^, 100 nC/μm^2^), the pitch point value corresponded to 13 nm. Figure 1 presents the obtained results using several techniques for morphological and topographical studies: A) optical microscopy, B) scanning electron microscopy (SEM), C) AFM, and D) correlative probe and electron microscopy (CPEM). The optical contrast of graphene placed onto SiO_2_/Si allows us to easily distinguish between its mono-, bi-, triple, and thicker flakes layers. The values (approx. 2.5 nm for a monolayer of graphene onto SiO_2_) measured using AFM, shown in the insets of Figure 1D, agree with the data obtained in studies presenting femtosecond laser thinning of graphene [28]. In addition to the region located below the baseline, we also observe an elevation in the central part of the exposed lines for all used dose values and dwell times (Figure 1C.i–iii), whose origin we discuss further in the subsequent section of the manuscript. The SEM and in situ AFM signals (Figure 1B, Figure 1C) are integrated into CPEM data, which yields additional insight into the substrate morphology. Apart from graphene etching, the morphological changes in the SiO_2_ surface are visible in AFM and CPEM results for lines that were extended beyond the flake area (see Figure 1D).

The experiments confirm that water-assisted FEBIE is a fast and direct technique to selectively remove graphene. The process must be optimized to prevent unnecessary defects and reduce the detrimental impact on the underlying substrate.

**Figure 2:**
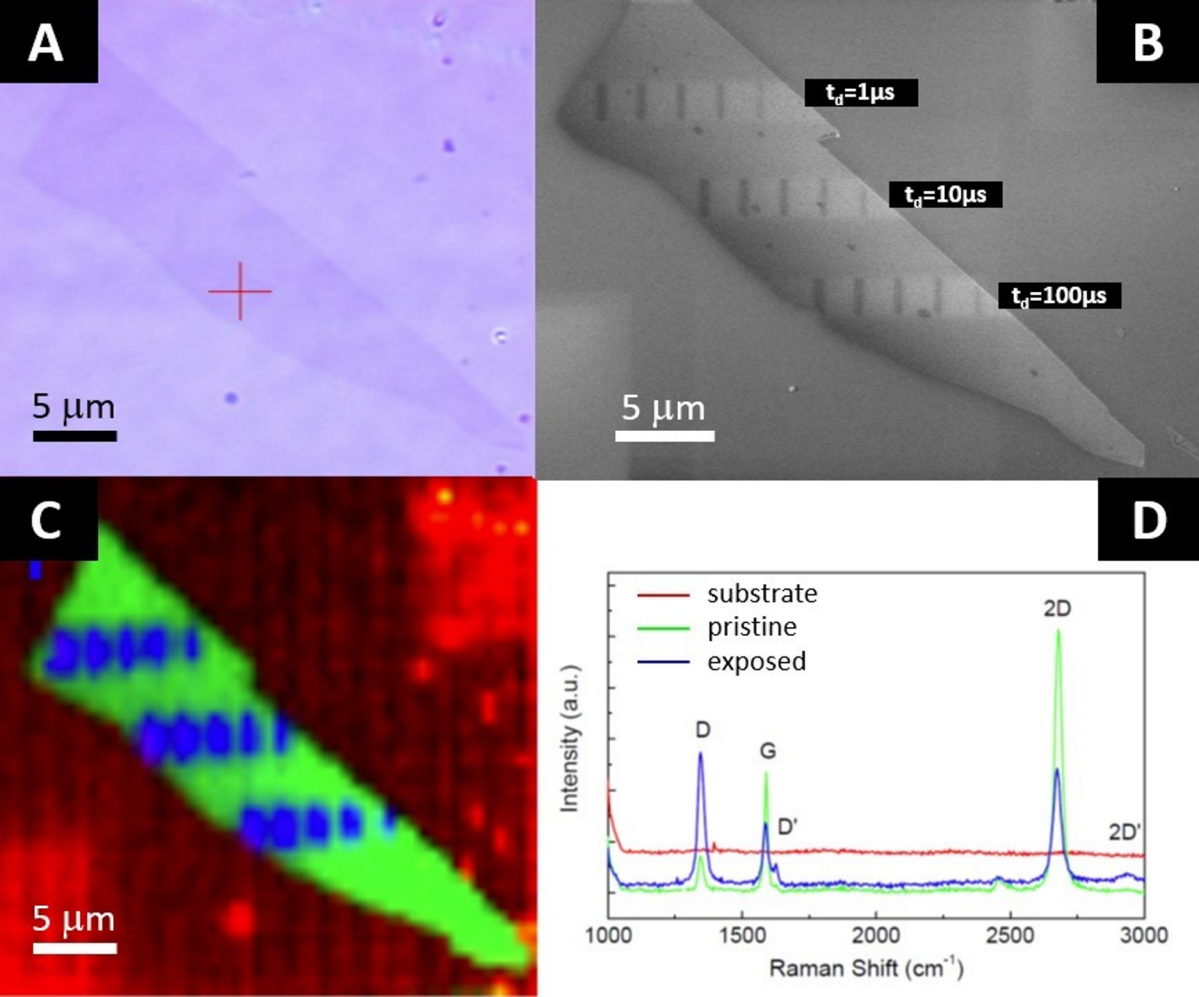
A) Optical microscope image of a graphene flake prior to patterning; B) SEM image of the same flake after the patterning process; C) Raman map according to the spectra in (D), revealing the substrate (red line), pristine (green line), and exposed regions (blue line).

The optical microscope image of the graphene flake before the patterning process is shown in Figure 2A. The size of the etched lines, estimated based on SEM measurements, is usually smaller than 50 nm (20 nm in the best case). However, due to the long residual time of the water molecules inside the SEM chamber, the collection of an image can further destroy the investigated material. Therefore, we performed a second series of experiments for a detailed analysis with Raman spectroscopy and AFM imaging without any additional exposure to the electron beam (apart from the etching process and short SEM inspection in HV mode using an extremely low current of 2.1 pA) prior to those measurements. As shown in Figure 2B, there is an apparent variation of the etched linewidth due to different processing times.

Three sets of lines with a length of 2 μm are etched in graphene, as can be seen in Figure 2B. Each horizontal set of lines corresponds to distinct dwell times (from 1 to 100 µs). Within each group, the dose decreases from left to right. The details of the exposure parameters, with an estimated beam size equal to 10 nm (FWHM), are summarized in Table 1. The results of Raman spectroscopy measurements are shown in Figure 2C and Figure 2D. This technique is not only sensitive to the number of graphitic layers in graphene but, more importantly, also to the number of defects, which can be estimated by the analysis of the D/G line intensity ratio [29–30]. Spectra of the underlying SiO_2_/Si substrate (red line), nonexposed (green line), and exposed graphene flakes (blue line) are collected in Figure 2D. Most of the graphene flake remains unaltered – the G/2D ratio is close to ½ as expected for single-layered graphene, and both peaks keep the Lorenzian shape. There are no features of amorphous carbon in the Raman spectra. Some defects can be elucidated from the noticeable presence of the D peak. It can be observed that the level of defects depends on whether graphene was or was not exposed to an electron beam. In the blue spectra, additional peaks (D’ and 2D’) are visible, and the intensity of the D peak is more pronounced. The Raman spectra were collected at each spot over the area containing the flake and the surrounding substrate. In the next step, based on the characteristic D/G intensity ratio, they were categorized into three groups. In this way, a map was constructed showing the correlation between the areas being exposed to the beam and the number of induced defects. The area where the defects are present in the vicinity of the etched lines increases with increasing electron dose. Due to the spatial resolution of the micro-Raman setup, we are unable to see the narrow etched lines with this microspectroscopic method.

**Table 1:** Exposure time and dose values for the etched lines.

Line no.	Length [μm]	Exposure time [s]	Charge [C]	Dose [nC/μm^2^]

D5	2	2	8 × 10^−9^	400
D4	2	1	4 × 10^−9^	200
D3	2	0.5	2 × 10^−9^	100
D2	2	0.25	1 × 10^−9^	50
D1	2	0.1	4 × 10^−10^	20

**Figure 3:**
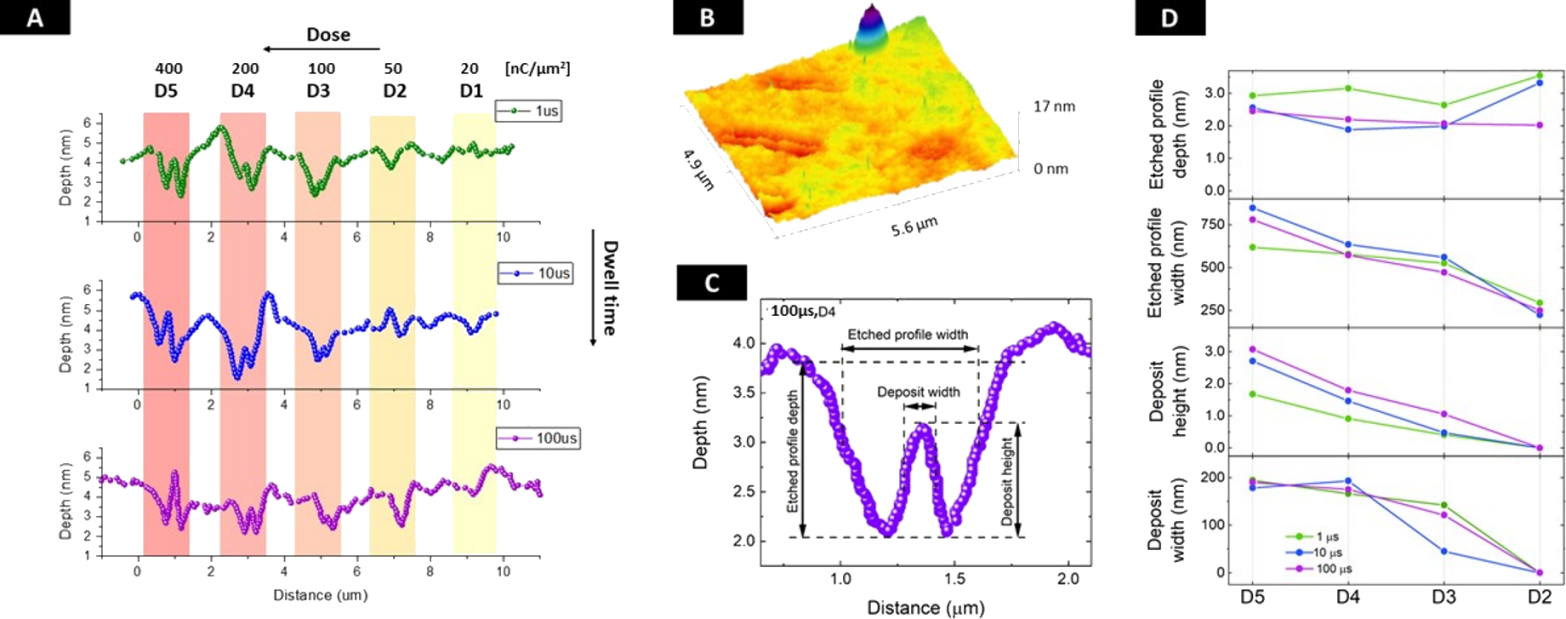
A) AFM profiles of the etched lines, with different dwell times (*t*_d_ = 1, 10, and 100 μs) and variable doses, the intensity of the color bars emphasizes the dose change. The parameters are summarized in Table 1. B) 3D AFM map of lines etched with 100 μs and doses D4, D3 indicating morphological changes in the SiO_2_ layer. C) Closeup of one of the trenches with main geometrical parameters of the etched features explained. D) Trench depth and width as well as the central deposit height and width as explained in C) obtained from AFM measurements for D2–D5 trenches.

The AFM technique allows for the determination of exact profiles of the etched lines, giving information about their depth and lateral size. The profiles, corresponding to three groups of patterns with given dwell times and varying electron doses, are presented in Figure 3. Already for the lowest electron dose D1 – 20 nC/μm^2^, one observes that graphene is effectively removed. From the profiles for the two lowest electron doses (D1 and D2), it is difficult to find any correlation between the profile shape and the dwell time. However, at dose D3, the protrusion in the central part is noticeable – its height increases with increasing dose and can be correlated to the chosen dwell times.

In order to examine the correlation between the electron dose and the results of the etching procedure, we performed careful data treatment and detailed calculations of the AFM measurements of the etched profile width and depth as well as central deposit/protrusion width and height (see Figure 3C for a visual explanation). The meaningful parameters are presented in Figure 3C. In the case of the depth of the profiles one cannot see any correlation with the dose. The width of the profile gets larger as the dose increases. Similarly, the central deposit is getting wider, and its height increases with an increasing dose.

**Figure 4:**
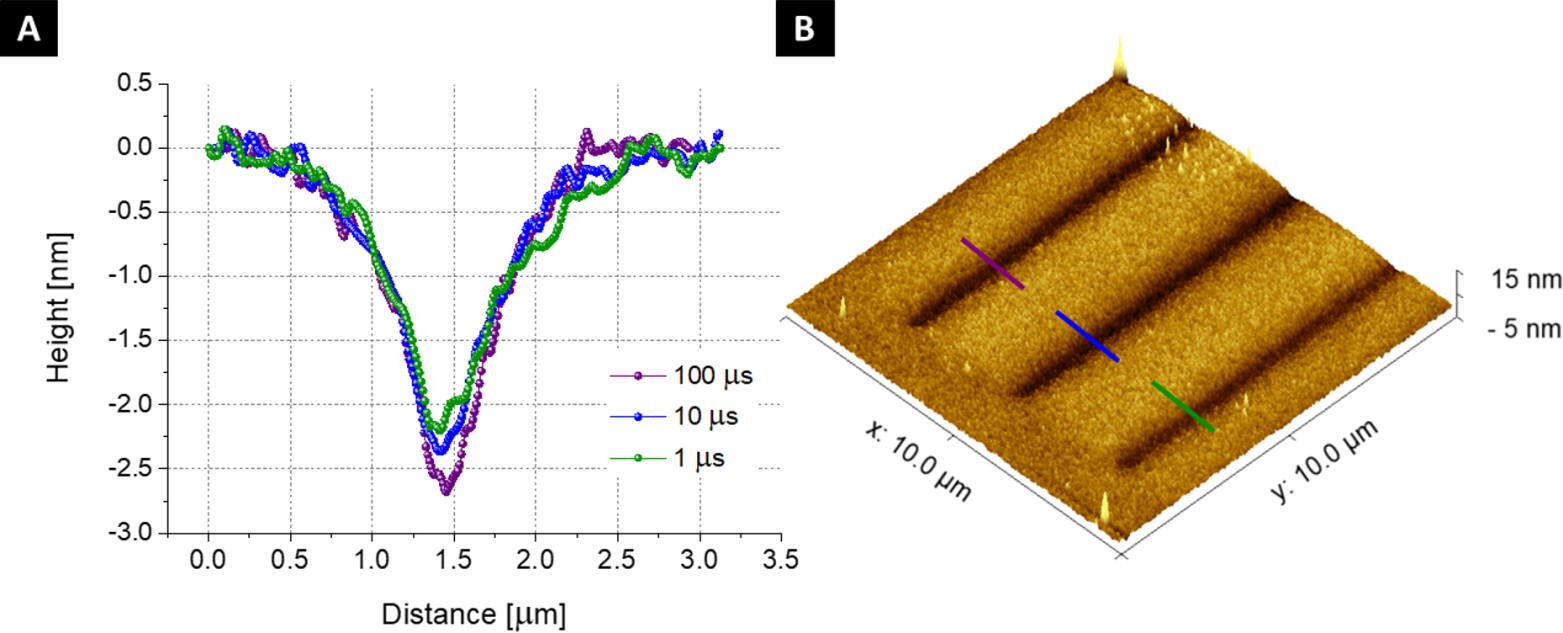
Results of in situ AFM measurements of the etched lines on the SiO_2_/Si substrate as a function of the dwell time: A) AFM profiles of lines etched with different dwell times (*t*_d_ = 1, 10, and100 μs); B) 3D AFM image of the SiO_2_ surface.

To assess the role of the electron beam during the presence of water on the SiO_2_ substrate, we performed in situ experiments with an AFM microscope (LitesScope^TM^) installed inside the SEM chamber, which allows measuring the profiles directly after electron beam exposure without contact with ambient air. The lines were directly patterned on the SiO_2_ substrate with the same dwell time values (1, 10, and 100 μs) and the highest dose D5. The profiles extracted from the AFM data are shown in Figure 4 and confirm the morphological changes introduced into the SiO_2_ substrate. In this case, the middle protrusion is not observed.

## Discussion

In general, the resolution of the FEBIE technique is related to both the beam size and the dwell time [22]. Our results show that the electron-beam-induced etching of graphene placed on a SiO_2_ substrate differs from the reported studies of etching carbon allotropes with water in terms of the dependence on dwell time and applied electron dose. The width of the AFM etch profiles is approximately 250 nm for dose D2, corresponding to the radius of backscattered electrons in Si (≈200 nm). The width scales up with the dose, and a correlation to the applied dwell time is evident. Notably, for the highest dose value, the width of the profile is the smallest with the shortest dwell time, as expected. In a similar way, the height of the central deposit/protrusion is codependent both on the dwell time and electron dose. The higher the dose, the more prounounced the effect of the dwell time on the profile. The height of the protrusion is scaled with the dose and depends on the dwell time. In order to verify whether the morphological changes observed by AFM outside of the SEM chamber are artifacts, we conducted an additional set of experiments under the same e-beam conditions in low-vacuum (LV) mode on SiO_2_/Si, but exposing large triangular patterns (5 × 10 μm^2^). The results, although not presented here, show similar behavior – for lower doses, we observe a depression, while for higher ones, we see an elevation. Their depth/height is correlated both with the dose and dwell time values. As the protrusion was not observed in AFM profiles measured in situ, we cannot exclude that it appears after taking out the sample and exposing it to air and ambient pressure. This effect, to the best of our knowledge, is firstly reported in this work and has not been mentioned in prior research on water-assisted FEBIE etching of graphene [17].

The central protrusion in the etch profiles is observed in ex situ AFM profiles at high doses in case of singular lines and large triangular patterns. Its origin is unlikely due to amorphous carbon co-deposition from the residual vapor impurities. Such transition from the deposition (FEBID) of amorphous carbon from the hydrocarbon contaminants to the water etching (FEBIE) at low vacuum mode (90 Pa of H_2_O) with increasing electron flux was previously observed by Toth et al. [23]. At a stationary exposure and current of 71 pA (lower electron flux), the authors observe a carbonous pillar-like deposit, and at a current of 245 pA (higher electron flux) the indent shape. This controllable switching between the hydrocarbons pinning and etching caused by increasing electron flux was explained using the continuum model. The model is based on the dissociation process of adsorbed molecules by electrons at a given process time. From the pressure *P* inside the SEM chamber one can calculate the number of impinging molecules by using the formula *J* = *PN*_A_/(2π*MRT*)^1/2^, given in the reference [31], where *N*_A_ is the Avogadro number, *M* is the molar mass of impinging molecules, *R* is the universal gas constant, and *T* is the temperature in absolute units. The water base pressure of 130 Pa corresponds to the water impinging rate of *J*_H2O_ = 4.7 × 10^6^ 1/nm^2^·s. Taking the size of the beam (FWHM) to be equal to 10 nm and the current to be equal to 4 nA, the electron flux at the center of the beam was calculated to be equal to *f*_e_ = 1.1 × 10^7^ nm^−2^·s^−1^. Based on the calculation results presented in this work, even at the lowest partial pressure of water (100 Pa) and at the lowest values of electron flux *f*_e_ ≈ 10^3^ nm^−2^·s^−1^ one should not observe any carbonaceous deposits, meaning that all hydrocarbons already deposited should be etched away with water. The parameters used in our experiments are even further from the switching point. Therefore, the protrusions in the middle of the line profiles, visible in Figure 3 for doses D3–D5, are unlikely due to the deposition of hydrocarbons.

The nanostructurizing process using electron beams and gas precursors was not widely recognized at the time when earlier studies regarding morphological changes in silica upon the electron irradiation were performed [24]. Therefore, neither of the electron-beam-induced changes into the SiO_2_ surface during the water purification of FEBID materials were taken into account.

The topographical changes in silica can be a consequence of removing Si and O atoms by OH^−^ groups, which are the product of adsorbed water molecules upon electron beam exposure. The related process of silica removal by hot water molecules at a high pressure has already been studied by several groups [32–35]. However, the direct mechanism has not yet been fully elucidated up to now [36]. In the studies of etching of thermally grown SiO_2_, reported in 1995 by Bakker and Hess [32], the authors claim that chemical reactions between Si atoms and OH^−^ groups lead to the formation of the volatile Si(OH)_4_ compound. A similar mechanism is proposed by Rosamila et al. [33] for O_2_-promoted water etching of Si surface, established on the observation that the etching rate is proportional to the concentration of OH^−^ groups. Those hydroxyl groups are also electron dissociation products of water molecules and thus could be actively involved in the SiO_2_ removal process.

Our results, showing topographical changes in SiO_2_ are partially consistent with former studies reported by Steven Kalceff et al. [24] of either swelling or deswelling of a silica substrate upon electron irradiation. Their findings on the volume decrease or increase of silica crystalline and amorphous phases were explained by the electromigration of oxygen atoms and densification of surface regions. Yet, taking into account the supplied data one cannot exclude the presence of water residues in the SEM chamber (and their different adsorption mechanisms on quartz or amorphous silica surfaces), which would be responsible for those topographical changes. This hypothesis could also be supported by the fact that OH^−^ groups can easily interact with the H-terminated surface of amorphous silica. However, they are less likely to oxidize quartz, where Si–O–Si bonds dominate on the surface. It is also consistent with other results, where oxygen gas was used for FEBIE of graphene and no etching of SiO_2_ substrate was detected by AFM.

Although our studies already untangle some phenomena accompanying the graphene etching using water-assisted FEBIE, the final interpretation of SiO_2_ morphological changes would require more efforts. For example, treating the subject separately with more sophisticated spectroscopy techniques to provide the data on chemical phase and types of Si–OH bonds on the surface and more advanced TEM cross-sectional analysis would be needed.

## Conclusion

The feasibility of water-assisted FEBIE, available on scanning electron microscopes operating in low-vacuum mode, makes the method very promising for prototyping various optical/electronic devices based on graphene. In this work, we showed that with certain precautions water-assisted FEBIE can be applied for such a nanopatterning process. The experimental data obtained with scanning Raman spectroscopy, correlative probe and electron microscopy, and in situ AFM measurements provide a comprehensive image of FEBIE etch profiles. In addition, the data reveal phenomena emerging from electron-induced interactions of adsorbed water molecules with SiO_2_, which have not been reported up to now. We observe that at low electron doses, the method provides a high spatial resolution and induces a low amount of defects in nonexposed areas of graphene, whereas at higher electron doses the resolution deteriorates and the number of defects significantly increases. Additionally, the etching process is accompanied by morphological changes in the microstructure of the substrate – likely occurring during exposure to the the ambient air and pressure. Complementary studies performed using both in situ and ex situ AFM reveal the modification in SiO_2_/Si substrate topography.

Our results are important not only for applications of water-assisted FEBIE to etching carbon allotropes and SiO_2_ materials but also in other fields. For example, where electron-driven reactions between H_2_O molecules and silica could occur, especially in the post-purification of granular materials composed of noble metals, such as Pt–C or Au–C which are directly deposited onto Si or SiO_2_ substrate.

## Experimental

### Sample preparation

For this experiment, we chose mechanically exfoliated graphene obtained with the standard Scotch^©^ tape method [1] from highly oriented pyrolytic graphite (HOPG) to take advantage of the lack of residues on top of the graphene layer and the lowest possible amount of defects. Cleaved flakes were deposited onto doped Si with a 285 nm thick SiO_2_ layer. Optical microscope evaluation allowed us to choose flakes that fitted our requirements the best in terms of lateral sizes and number of graphitic layers (optical contrast abruptly changes from monolayer to the bilayer, triple-layer, or HOPG) [37]. The same substrates of separate studies of topographical changes induced in the SiO_2_ upper layer were cleaned for ten minutes in an ultrasonic bath with acetone, isopropanol, and O_2_ plasma before being placed in the microscope chamber.

### Graphene and SiO_2_/Si etching

The samples were placed inside the SEM-Versa 3D (FEI), which also operates at LV mode. The chamber was pumped down to a pressure below 3 × 10^−4^ Pa. Next, SEM was set into the LV mode to fill the chamber with water vapor to the pressure of 130 Pa during the etching processing. The electron gun extraction voltage was then set to 5 keV, and the electron beam current to *I*_B_ = 4 nA, providing an approximate lateral beam size of 10 nm. The patterned structures consisted of three groups of five single-pixel lines (SPL). The length of each SPL was fixed and set to 2 µm. The delivered dose was controlled by the processing time of each line (2 s, 1 s, 500 ms, 250 ms, and 100 ms). The dwell time (*t*_D_) (i.e., the time while the electron beam dwells at each pixel) was varied between the groups (1 µs, 10 µs, 100 µs) to check for optimal process parameters.

### Raman spectroscopy

Raman spectroscopy allows for the examination of various properties of graphene sheets offering a nondestructive approach. We used a confocal Raman Alpha 300 M+ from WITec, which combines a Raman spectrograph with a confocal microscope. A laser with a 532 nm wavelength, spot size of 1 μm, and power fixed at 1 mW was used to avoid sample heating. The confocal microscope gives a higher lateral resolution than conventional optical microscopes, leading to enhanced quality signals. The equipment has an automatic motorized sample positioner in the x-, y- and z-directions that allows 2D and 3D mapping. For our study, we performed 2D mapping with a lateral step size of 1 µm. The WITec software was used to process these spectra selecting those with similar characteristics, allowing us to distinguish between irradiated and nonirradiated areas of the graphene layer and evaluate the etching results.

### Atomic force microscopy

Precise surface analysis of etched structures can be performed by AFM. We used a unique AFM LiteScope from NenoVision s.r.o., a compact and powerful AFM setup capable of integrating into a scanning electron microscope. The LiteScope is equipped with a CPEM technology for simultaneous acquisition of AFM and SEM signals, enabling an efficient and complex surface analysis. The AFM-in-SEM approach allows for in situ analysis. Thus, the graphene could be modified and immediately measured by AFM without changing the environment. The analysis was performed by using the tapping mode and the Akiyama probe (Nanosensors). Data shown in Figure 4 were measured with the following parameters: scan speed of 20 µm/m, scan range of 20 µm × 20 µm, and image resolution of 512 × 512 pixels. The resonance frequency of the particular AFM probe was 43 kHz. The acquired data were post-processed using the Gwyddion software from the Czech Metrology Institute.

The ex situ measured AFM profiles of SiO_2_ substrate after the processing with e-beam were taken using the Bruker Dimension ICON XR PeakForce in tapping mode in air with image resolution of 258 × 258 pixels and then post-processed with Gwyddion.

## Data Availability

The data that supports the findings of this study is available from the corresponding author upon reasonable request.
